# Report of Committee on Nomenclature of Hog Cholera and Swine Disease

**Published:** 1894-11

**Authors:** 


### Report of Committee on Nomenclature of Hog Cholera and Swine Disease, 31st Annual Meeting, United States Veterinary Medical Association.

Philadelphia, Pa., Sept., 1894.

In view of the fact that various names have been and are given in foreign countries to the diseases usually called hog cholera by veterinarians in this country, and that some European investigators have adopted the name of swine plague or American swine plague, employed by F. Billings to designate the disease commonly called hog cholera in the United States, and that not a little confusion is caused by this diversity of nomenclature. Your Committee recommends that, at least until some international agreement is reached, the ordinary usage be retained, and that the name hog cholera continue to be applied to that epizootic disease of swine, which has now been proven to be caused by the bacillus called the hog cholera bacillus in the Reports of the Bureau of Animal Industry since the Report for 1886. This bacillus has been shown to be identical with that called swine plague bacillus by F. Billings. The American disease, hog cholera, has been shown to be identical with the disease usually called swine fever in England, swine pest in Scandinavian countries, and pneumo-enteritis of swine in France, and is distinct from the German disease, schweine seuche.

The name, swine plague, employed by the Bureau of Animal Industry to designate an affection caused by the swine plague bacterium of Salmon and Smith, may continue to be so used, pending further investigation.

The disease of swine, which is of chief practical importance to veterinarians in this country as a widely spread and fatal epizootic, is hog cholera. The swine plague bacterium appears to be identical in its morphology and cultured properties with the schweine seuche bacterium, and if this identity be established beyond doubt it will be a further reason for the continuance of the term swine plague, as applied to a disease very prevalent, at least in some countries.

A. W. CLEMENT, *Chairman.*

JAMAICA PLAINS, Sept. 17th, 1894.

DEAR CLEMENT: I have just received your report, so late that you will doubtless have read it before you hear from me. I believe I have already given you my views on the subject, so it is needless for me to tell you that I do not altogether agree with you in what you say. First, the term, hog cholera, means nothing, and is simply an every-day, unscientific name given by farmers. What we have called hog cholera is the only real swine plague we have in this conntry, and we, as public educators, should try to bring about the adoption of a proper nomenclature for this disease. Secondly, what the Bureau of Animal Industry calls swine plague is not really a swine plague, because sheep, lambs, calves, and probably other cattle, and very likely horses, may also have it. This feature of the disease you do not even mention in your report. The only feature of your epistle I agree to is the correctness of the scientific facts as you state them, and the expression of obligation to Prof. Welch for his kind interest in the work.

Your report as a Committee must go in without my signature as a member, for the reasons stated.

Very sincerely yours,

AUSTIN PETERS.

